# Multi-Response Optimization of Ultrafine Cement-Based Slurry Using the Taguchi-Grey Relational Analysis Method

**DOI:** 10.3390/ma14010117

**Published:** 2020-12-29

**Authors:** Shuai Zhang, Weiguo Qiao, Yue Wu, Zhenwang Fan, Lei Zhang

**Affiliations:** 1 Shandong Key Laboratory of Civil Engineering Disaster Prevention and Mitigation, Shandong University of Science and Technology, Qingdao 266590, China; zhangshuai92@sdust.edu.cn (S.Z.); wysdkd2019@sdust.edu.cn (Y.W.); fanzw94@sdust.edu.cn (Z.F.); zhanglei96@sdust.edu.cn (L.Z.); 2 College of Civil Engineering and Architecture, Shandong University of Science and Technology, Qingdao 266590, China

**Keywords:** ultrafine cement-based slurry, colloidal nanosilica, sodium sulfate, orthogonal test, Taguchi-Grey relational analysis, microstructure

## Abstract

The grouting technique is an important method in underground engineering that prevents water seepage and reinforces fractured rock mass. In this research, ultrafine cement-based grouting material, including ultrafine cement (UC), ultrafine fly ash (UFA), polycarboxylate superplasticizer (SP), colloidal nanosilica (CNS), sodium sulfate solution (SS) and water, was developed. The flow time, viscosity, bleeding, setting time and uniaxial compressive strength of the UC-based slurry were measured by orthogonal experiments, and the optimal mix proportion of the UC-based slurry was obtained based on the Taguchi-Grey relational analysis method. Microstructure analysis of the UC-based slurry was conducted using scanning electron microscopy (SEM) and mercury intrusion porosimetry (MIP) tests. The results showed that the Bingham model could provide a satisfactory description of the rheological properties of the UC-based slurry. The addition of CNS and SS could promote the hydration of the UC-based slurry and improve the microstructure of the hardened slurry, thereby increasing the strength of the hardened slurry. The optimum ratio for the UC-based slurry was water/solid (W/S) ratio of 1.0, and the contents of UFA, SP, CNS and SS by mass of UC were 40%, 0.2%, 4% and 4%, respectively.

## 1. Introduction

With the increasing shortage of shallow coal resources, the depth of coal mining has been gradually increasing. Under the condition of deep high ground stress and high hydraulic pressure, water seeps from the microfractures in the rock mass, resulting in large area water seepage problems in surrounding rock, which seriously affects the safety and stability of the construction, operation and maintenance of tunnels and metros [1,2,3]. In recent years, grouting technique has been widely adopted for the prevention of geological disasters, for example, water leaks, floor heaves and landslides in deep roadways, tunnels, slopes and dams [4]. In grouting technology, a suitable grouting material is the key factor affecting the success of the grouting. Grouting materials mainly include cement-based slurries, chemical slurries and organic-inorganic composite slurries, among which cement-based slurries are most widely used because of their lower cost and reduced harm to the environment. However, it is difficult to inject ordinary Portland cement slurries into fine sand or microfractures [5]. In recent years, ultrafine cement (UC) slurry has been rapidly developed and is widely used to prevent the water seepage problem in underground engineering [6,7,8].

Fly ash is the main component of the solid waste discharged from thermal power plants, and fly ash output has shown an increasing trend in China. Fly ash has many adverse effects on people’s lives, such as soil pollution, water pollution, air pollution and so on. In recent years, fly ash has been widely used as a mineral additive in cement and concrete to improve the properties [9,10]. The addition of fly ash not only improves the flowability of fresh slurry and the durability of hardened slurry but also improves later-age strength and reduces the costs. Li et al. [7] studied the rheological properties of ultrafine-cement-based pastes, and the results showed that the addition of fly ash improved the fluidity of fresh pastes. Shafigh et al. [11] investigated the mechanical properties of high-volume fly ash concrete, and the test results indicated that the addition of fly ash improved the later compressive strength of concrete that had been cured for more than 56 days. However, the replacement of cement by fly ash shows some disadvantages, such as a decrease in the early-age strength and prolongs the setting time [12]. Therefore, many researchers have attempted to find ways to solve the defects caused by a large amount of fly ash, such as adding nano-scale sized particles (nano-SiO_2_, nano-CaCO_3_, nano-MgO) to high-volume fly ash concrete [13,14,15].

Sato and Beaudoin [16] studied the effect of the addition of nano-CaCO_3_ in the hydration process of cementitious materials including fly ash and slag. They reported that nano-CaCO_3_ can clearly promote the early stage of hydration and also improve the modulus of elasticity. The investigation by Seifan et al. [17] reported that the addition of 5% nano-silica increased the 28-day compressive strength of geopolymer mortar containing different amounts of fly ash. Shaikh et al. [18] studied the addition of nano-silica on the performance of high-volume fly ash mortar, and the test results showed that the addition of 2% nano-silica was the optimal content in the high-volume fly ash mortar wherein it resulted in the highest compressive strength both at an early and later age. Xi et al. [19] developed a high-performance cement-based material containing nano-silica and fly ash cenosphere and studied the physical and mechanical properties of this material. They found that the addition of nano-silica decreased the porosity and improved the compactness of the material system due to its high pozzolanic reactivity. Zhang et al. [20] investigated the addition of colloidal nano-silica on the mechanical properties of UC-based slurries containing different fly ash contents. The test results showed that the adverse effect of fly ash on the early compressive strength of the hardened slurry was negated by the addition of colloidal nano-silica. According to these studies, the addition of nanoparticles can be inferred to improve the physical and mechanical properties of cement-based materials containing fly ash.

In addition to nanoparticles, many researchers have found that some chemical additives, such as sulfate activator, can also stimulate the early activity of fly ash in the cement-fly ash system. Sulfate activator can react with alumina to form ettringite (Aft), which has a remarkable early strength; this is achieved by promoting the pozzolanic reaction of fly ash. Shi and Day [21] studied the addition of sodium sulfate on the compressive strength of the lime-fly ash paste. The test results showed that the addition of sodium sulfate improved the early compressive strength of lime-fly ash paste due to the formation of Aft at an early age. Bui et al. [22] also investigated the effect of sodium sulfate on the mechanical properties of fly ash cement paste. They found that the addition of sodium sulfate promoted the pozzolanic reaction of fly ash, which improved the early compressive strength of the hardened paste. Based on the research above, the addition of sodium sulfate can be inferred to be beneficial to the early strength development of a fly ash cement system.

Although the influences of a one-part colloidal nanosilica and sodium sulfate on the mechanical properties of fly ash cement slurry have been investigated, the mechanisms of the combination of colloidal nanosilica and sodium sulfate on the performance of cement-based slurries have not been revealed. Therefore, the aim of this paper is to develop a new type of UC-based slurry for microfracture grouting and to explore the effects of admixtures on the performance of this ultrafine cement-based slurry. In this study, UC-based slurry, mainly containing UC, UFA, SP, CNS, SS and water, was developed. Based on the orthogonal test method, the studied factors included the water/solid (W/S) ratio, UFA content, SP content, CNS content, SS content, and the four levels of the five factors having strongest impact on the performance of the slurry were selected. A rheological model, the flow time, viscosity, bleeding, setting time, compressive strength and microstructure of the UC-based slurries were studied. The optimal mix proportion of the UC-based slurry was obtained based on the Taguchi-Grey relational analysis method.

## 2. Materials 

**Figure 1:**
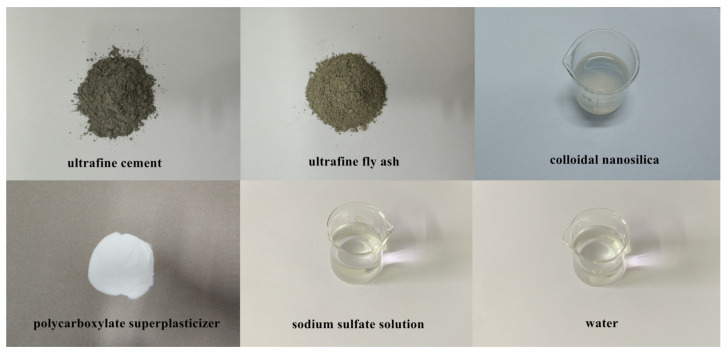
Composition materials of the designed UC-based slurry.

**Figure 2:**
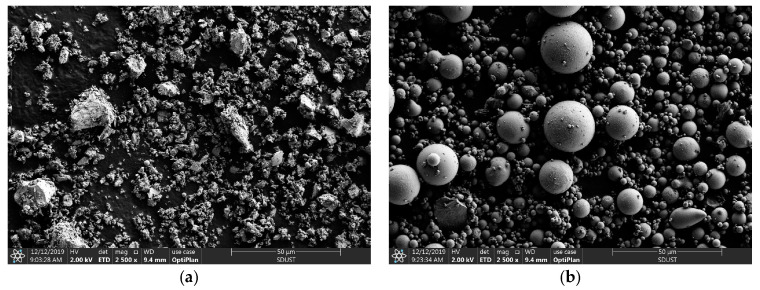
SEM photographs of the UC and UFA: (**a**) UC; (**b**) UFA.

**Figure 3:**
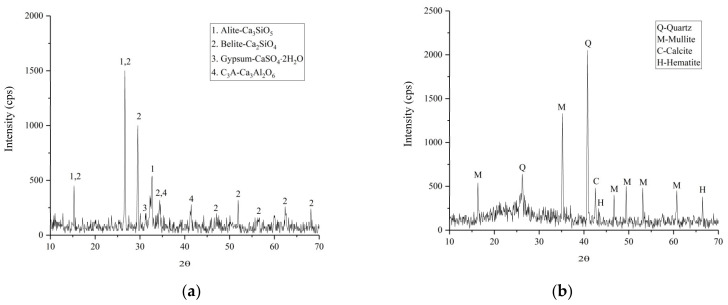
XRD analysis of the UC and UFA: (**a**) UC; (**b**) UFA.

The UC and UFA were produced by superfine grinding of P.O 42.5 Portland cement (Shandong Shanshui Cement Group Limited, Jinan, China) and class F fly ash (Shandong Shunke Building Materials Technology Co., Ltd., Longkou, China), respectively, and the strength characteristics of the cement and fly ash were confirmed to adhere to Chinese standards. The compositions of the UC and UFA are listed in Table 1. Commercially available colloidal nanosilica from Guangzhou Suize Environmental Protection Technology Co., Ltd. (Guangzhou, China) was used in the experiments. The basic properties of the CNS were obtained by the manufacturers and are presented in Table 2. In this study, a 40% sodium sulfate solution (SS) (Shandong New Building Materials Technology Co., Ltd., Qingdao, China) was used as an accelerator for the slurry. Additionally, polycarboxylate superplasticizer (solid) (Shandong New Building Materials Technology Co., Ltd., Qingdao, China) was selected as a high-range water reducer to prepare the cement slurry. Tap water was used to prepare the UC-based slurry. The composition materials of the designed cement slurry are shown in Figure 1. Scanning electron microscopic (SEM) photographs of the UC and UFA are shown in Figure 2 and illustrate the characteristic morphology and shape of the UC and UFA powders. The shape of the UFA particles was spherical and smooth, while the shape of the UC particles was angular and irregular, as shown in Figure 2. The X-ray diffraction (XRD, Rigaku Utima IV, Rigaku Corporation, Tokyo, Japan) results of the UC and UFA are presented in Figure 3. The XRD spectra of the UC indicate that the UC is mainly composed of alite and belite, as shown in Figure 3. The XRD results of the UFA showed that the UFA is mainly composed of an amorphous phase and some crystalline phase, such as quartz and mullite. To reduce the interference caused by temperature, all samples were placed in a laboratory with a temperature of 20 °C.

## 3. Experimental Methods

### 3.1. Orthogonal Arrays and Taguchi-Grey Relational Analysis Method

An orthogonal experimental design adopting the Taguchi method was used in this research. Based on the orthogonality, orthogonal experimental design selects some representative points from the comprehensive experiment, and these points have uniform dispersion and uniformity [23]. In the orthogonal tests, the factors are the parameters that affect the properties of slurry, and the level in the orthogonal tests refers to the specific conditions for each factor to be compared. Therefore, in this study, an orthogonal test was conducted to study the multi-objective parameters of the raw material mix proportions to find the optimal scheme. The Taguchi design method converts the experimental results to a signal-to-noise (S/N) ratio. The value of S/N ratio indicates the dispersion around the desired results and includes three types of performance characteristics: lower-the-better, nominal-the-better and higher-the-better [24]. For example, a shorter setting time, a lower viscosity, flow time and bleeding and a higher fluidity and strength are required in UC-based slurries. The S/N ratios of the three characteristics can be calculated using Equations (1)–(3):

(1) $$\mathrm{Lower}-\mathrm{the}\;\mathrm{better:}\;S/N=-10\log_{10}\left(\frac{1}{n}\sum_{i=1}^{n}y_{i}{}^{2}\right)$$

(2) $$\mathrm{Nominal}-\mathrm{the}\;\mathrm{better:}\;S/N=-10\log_{10}\left(\frac{1}{n}\sum_{i=1}^{n}{\left(y_{i}-y_{0}\right)}^{2}\right)$$

(3) $$\mathrm{Higher}-\mathrm{the}\;\mathrm{better:}\;S/N=-10\log_{10}\left(\frac{1}{n}\sum_{i=1}^{n}y_{i}{}^{2}\right)$$

where *y_i_* is the obtained value of *i*th test for *j*th response, and *n* is the number of replications.

The experimental procedures of the Taguchi-Grey relational analysis method are described as follows:

Step 1: Select the orthogonal test parameters and their corresponding levels.
Step 2: Select the suitable orthogonal table based on the Taguchi method, and arrange the orthogonal test parameters and their corresponding levels.
Step 3: Carry out the tests on UC-based slurries according to the Taguchi experimental design method.
Step 4: Calculate the S/N ratio of the orthogonal test results using the corresponding equation given by Equations (1)−(3) and analyze the variance in the S/N ratio.
Step 5: Normalize the S/N ratios of each response using Equations (4)–(6):

(4) $$Z_{ij}=\frac{\max(y_{ij})-y_{ij}}{\max(y_{ij})-\min(y_{ij})};i=1,2,\ldots ,m;j=1,2,\ldots ,n$$

Equation (4) is used to normalize the S/N ratio for lower-the better:

(5) $$Z_{ij}=\frac{\left|y_{ij}-\mathrm{Target}\right|-\min(\left|y_{ij}-\mathrm{Target}\right|)}{\max(\left|y_{ij}-\mathrm{Target}\right|)-\min(\left|y_{ij}-\mathrm{Target}\right|)};i=1,2,\ldots ,m;j=1,2,\ldots ,n$$

Equation (5) is used to normalize the S/N ratio for nominal-the better:

(6) $$Z_{ij}=\frac{y_{ij}-\min(y_{ij})}{\max(y_{ij})-\min(y_{ij})};i=1,2,\ldots ,m;j=1,2,\ldots ,n$$

Equation (6) is used to normalize the S/N ratio for higher-the better.

Step 6: Calculate the mass loss function using the following equation:

(7) $$\Delta =\left(Z_{\max}-Z_{ij}\right);i=1,2,\ldots ,m;j=1,2,\ldots ,n$$

Step 7: Calculate the grey relational coefficient (GRC) with the following equation:

(8) $$GRC_{ij}=\frac{\min\left(\Delta \right)+\lambda \max\left(\Delta \right)}{\Delta_{ij}+\lambda \max\left(\Delta \right)};i=1,2,\ldots ,m;j=1,2,\ldots ,n$$

Step 8: Calculate the grey relational grade (GRG) with the following equation:

(9) $$GRG_{i}=\sum_{j=1}^{n}\varphi_{j}GRC_{ij};i=1,2,\ldots ,m$$

where:

*y_ij_* is the S/N ratio value of *i*th experiment for the *j*th response;
Z*_ij_* is the *i*th normalized S/N ratio value for the *j*th response;
Δ*_ij_* is the difference between the optimum value of the normalized S/N ratio and the *i*th normalized S/N ratio value for the *j*th response;
*λ* is the identification coefficient that ranges from 0 to 1, and *λ* is generally set as 0.5;
*φ_j_* is the normalized nonnegative coefficient assigned to the jth response, and the sum of all *φ_j_* is 1. All the responses (characteristics) considered in this research are equally weighted.

In this research, UC-based slurry mainly included UC, UFA, CNS, SP, SS and potable water. Based on the Taguchi experimental design method, five factors, i.e., W/S ratio (A), UFA content (B), SP content (C), CNS content (D) and SS content (E), were selected, and their corresponding levels are shown in Table 3. According to the Taguchi method, the L16(4^5^) orthogonal test scheme used for the experiment is shown in Table 4.

### 3.2. Experimental Methods of the Specimens

In this study, the particle size, viscosity, flow time, bleeding, setting time, and uniaxial compressive strength of the UC-based slurry were evaluated. In this research, at least three specimens were measured for each experiment to ensure the reliability of experiment results. The grain size distributions were measured by a laser particle size analyzer (LS900, OMEC Company, Zhuhai, China). The viscosity of the fresh slurry was measured using a SNB-2 coaxial cylinder rheometer (Shanghai Ping Xuan Scientific Instrument Co., Ltd., Shanghai, China) with viscosity measurements ranging from 1 to 6 × 10^6^ mPa·s. The flow time is defined as the time required for 946 ml of the fresh slurry to move through a Marsh funnel [25]. More specifically, the Marsh funnel had an internal diameter of 4.8 mm, and the flow time for 946 mL water was 26 ± 0.5 s. The bleeding of the fresh slurry was measured by a sedimentation test and calculated by *ΔV*/*V*_0_, where *V*_0_ is the initial volume of fresh slurry and *ΔV* is the volume of the bleeding water at 2 h [26]. The setting time of the fresh slurry was measured by a Vicat needle tests based on GB/T 1346-2011. To measure the uniaxial compressive strength of the hardened slurry, cylindrical samples (50 mm in diameter and 100 mm high) were prepared and cured in a humid room (20 ± 2 °C and 95% R.H.) for 7 and 28 days. The compressive strength of slurry was tested by a TAW-2000 rock mechanics (Changchun Chaoyang Test Instrument Co., Ltd., Changchun, China) servo-controlled testing system with a loading rate of 0.5 mm/min.

To study the morphology of the UC-based slurry hydration products, SEM (Apreo, FEI Corporation, Hillsboro, OR, USA) was used to observe the microstructure of the hydration products. MIP measurement was used to quantitatively study the porosity and pore size distribution of the specimens. The specimens were tested by a Pore Master series Quantachrome instrument (Micromeritics Instruments Corporation, Norcross, GA, USA) with a maximum pressure of 414 MPa and a contact angle of 130°. Before the MIP test commenced, samples were dried at approximately 110 °C for 24 h to a constant weight.

## 4. Results and Discussion

### 4.1. Grain Size Analysis

**Figure 4:**
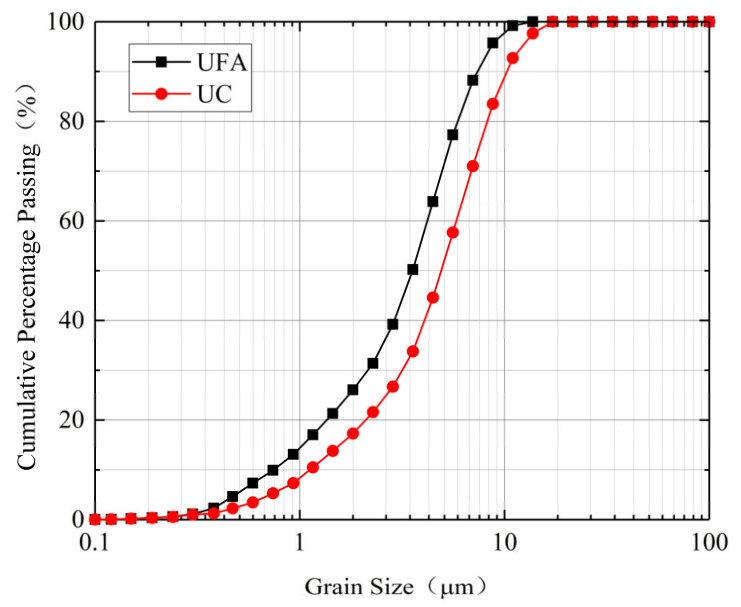
Particle size distributions of UC and UFA.

To better study the rheological and mechanical properties and the groutability of a microfine-cement-based slurry, it is necessary to analyze the particle size distribution [27]. The grain size distributions of the UC and UFA were obtained using a laser diffraction technique, and the results are presented in Figure 4. Characteristic grain sizes and specific surface of UC and UFA are listed in Table 5. In terms of the grain size, both the UC and UFA can be considered as ‘microfine’ since the grain size value meets the requirements of EN 12715 (specific surface > 800 m^2^/kg and *d*_95_ < 20 µm). A primary evaluation of the fracture groutability was made by calculating the ‘groutability ratio’ which is defined as *R* = *D*_p_/*d*_max_, where *D*_p_ (unit: µm) is the aperture of the fracture and *d*_max_ (unit: µm) is the maximum diameter of the cement particles [28,29]. Grouting is considered possible if *R* > 3 and satisfactory if *R* > 5 [28]. This is because the rock fracture groutability of a slurry is reduced due to the agglomeration phenomenon. In this study, *R* = 300 µm/17.46 µm = 17.2; thus, satisfactory permeation can be achieved.

### 4.2. Analysis of the Orthogonal Test Results

#### 4.2.1. Rheological Model of the UC-Based Slurry

**Figure 5:**
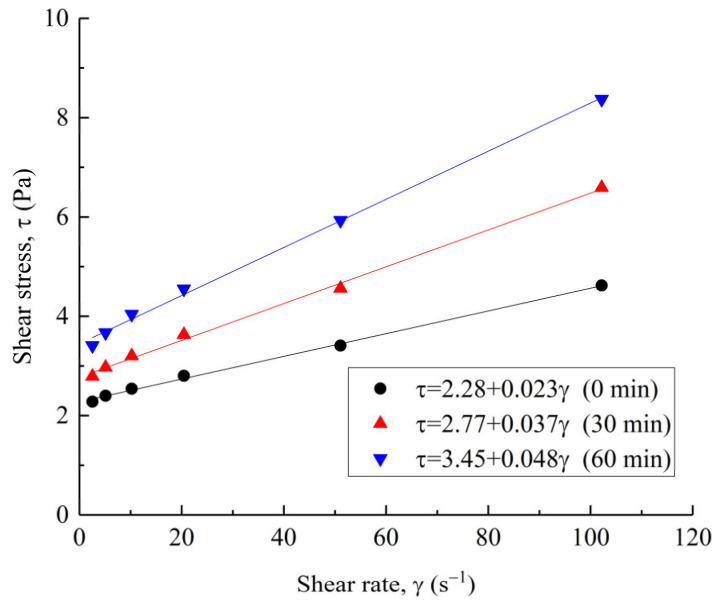
Typical rheological curves of fresh UC-based slurry (G1).

The rheological model can describe the relationship between the shear stress and shear rate and refers to the evaluation of the rheological properties of UC-based slurry [30,31]. Compared with ordinary Portland cement-based grouting materials, the viscosity and shear stress requirements for microfracture grouting are different. In the context of microfracture grouting materials, a UC-based slurry should have a low viscosity and shear stress to achieve and maintain the desired levels of flowability and groutability in a microfracture. Some typical rheological curves of fresh UC-based slurry (G1) are presented in Figure 5. 

It can be observed from Figure 5 that the rheological curves (down-curves) of fresh slurry are almost a straight line intersecting the ordinate axis or have an initial fluctuation followed by a straight line. A similar result was also reported by Pantazopoulos et al. [32] and Zhang et al. [26]. Therefore, the relationship between the shear rate and shear stress can be regarded as linear, which is well described by the Bingham model. The rheological parameters, yield stress and plastic viscosity were obtained by the Bingham model:

(10) $$\tau =\tau_{0}+\eta \gamma$$

where *τ*, *τ*_0_, *η*, and *γ* are the shear stress (Pa), yield stress (Pa), plastic viscosity (Pa·s), and shear rate (s^−1^), respectively. After fitting analysis, the values of the plastic viscosity, yield stress and correlation coefficient, *R*^2^, for the slurry immediately after preparation are shown in Table 6.

As presented in Table 6, the correlation coefficients were equal to 0.99 in all cases, indicating the Bingham model fitted the experimental data satisfactorily. Overall, the yield stress and plastic viscosity of the fresh slurry increased with the test time. For fresh slurry G1 (0 min to 60 min), the yield stress ranged from 2.28 to 3.45 Pa (increase of 51%) and the plastic viscosity ranged from 23 to 48 mPa·s (increase of 109%). The effect of the test time is more significant on the plastic viscosity rather than on the yield stress. The increasing effect on the rheological parameters can be due to the fact that, with an increase in the test time, the hydration reaction of the fresh slurry begins, the free water in the slurry decreases and the sediment increases; the slurry gradually become viscous, and the friction resistance inside the slurry also increases; therefore, the yield stress and plastic viscosity increase [33].

#### 4.2.2. Flow Time and Apparent Viscosity of the UC-Based Slurry

**Figure 6:**
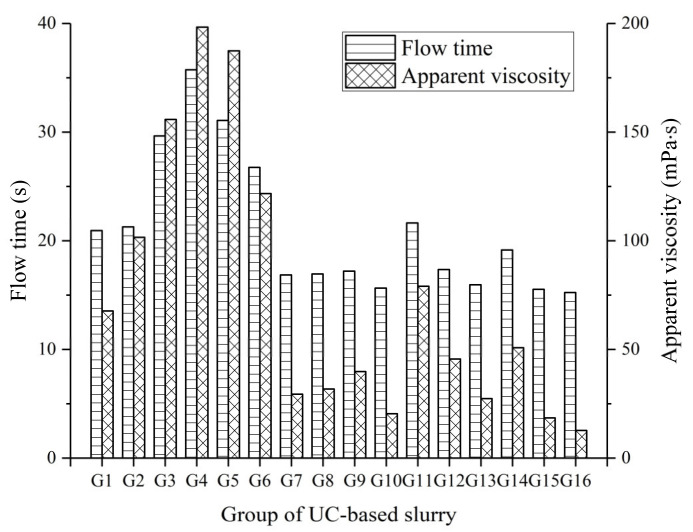
Flow time and apparent viscosity of the UC-based slurry.

The flow time and apparent viscosity are important parameters when evaluating the fluidity or workability of fresh slurry, and low viscosity or high fluidity can improve the groutability and spreading ability for microfractures. The flow time and apparent viscosity results of fresh slurry (G1 to G16) are presented in Figure 6.

As indicated in Figure 6, the flow time and apparent viscosity of slurry gradually increased due to the increase of the CNS and SS content in the mix, while these parameters decreased with an increase in the W/S ratio. Among all the UC-based slurries, the mix G4 (1.0 WS ratio, 40% UFA, 0.2% SP, 4% NS, and 4% SS) had the highest flow time (35.73 s) and apparent viscosity (198.3 mPa·s). This behavior can be explained by the following reasons: (i) the particle size of the CNS is smaller than that of cement, resulting in a significant decrease in the space between particles and, hence, increasing the chance of contact between particles; (ii) the addition of CNS causes the specific surface area of the particles to unavoidably increase, leading to more water wetting the particles surfaces. Therefore, the increase in the friction and flow resistance causes an increase in the flow time and apparent viscosity of the fresh slurry. Roshani and Fall [34] also reported similar results. In addition, group G16 showed both lower flow time and apparent viscosity (15.24 s and 15.7 mPa·s), which can be attributed to the dominant effect of the higher W/S ratio (1.6) followed by the ball effect of the UFA and lower SS content (2%) in the mix.

**Figure 7:**
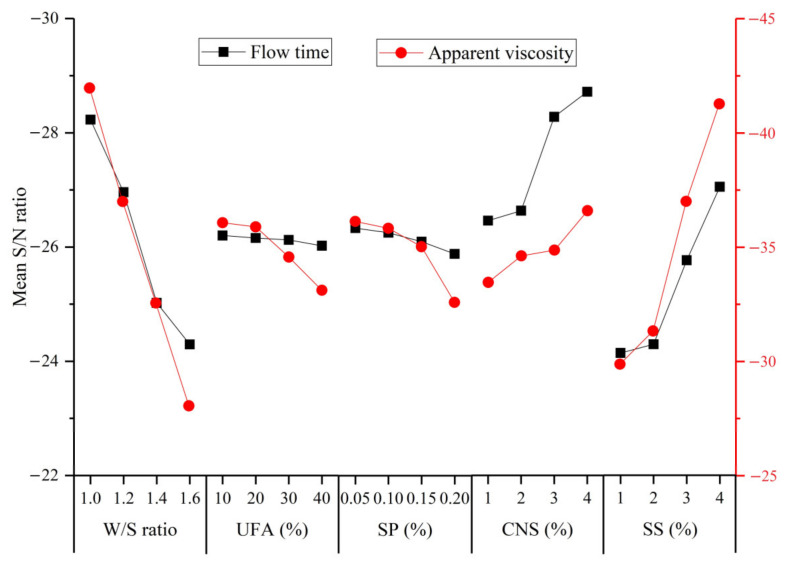
Mean S/N ratio values of flow time and apparent viscosity of the UC-based slurry for different factors.

The S/N ratios given by the flow time and apparent viscosity and calculated by Equation (1) are shown in Table 7. The mean S/N ratio for a given response with regard to a given factor level is defined as the average value of the S/N ratio of that response for the slurry prepared with that factor level. The mean S/N ratio values of the flow time and apparent viscosity of the UC-based slurry for different factors are presented in Figure 7.

As indicated in Figure 7, the mean S/N ratio values of the flow time and apparent viscosity decreased significantly when the W/S ratio of the slurry increased from 1.0 to 1.6. The reason for this observation can be ascribed to the presence of more free water, which decreases the friction force inside the cement slurry. The mean S/N ratio also decreased with an increase in the UFA and SP content indicating lower flow time and apparent viscosity of the UC-based slurry. This positive effect of the UFA on the flow time and apparent viscosity can be ascribed to the following reasons: (i) the UFA particles are mostly spherical vitreous bodies with a smooth surface and improve the lubrication effect in fresh slurry, resulting in a reduction in the friction between the particles; and (ii) the finer UFA particles can fill the voids between the cement particles and release more free water to form a water film covering the surface of the particles [35]. The decrease in the flow time and apparent viscosity with an increase in the SP content can be ascribed to the following mechanisms: the addition of SP prevents the close contact between particles, which can be attributed to the fact that the combination of static electricity and spatial repulsion can eliminate the agglomeration of cement particles; the adsorption surface of the SP can destroy the flocculation structure produced at rest, and hence, the fluidity of the fresh slurry can be improved. However, the flow time and viscosity increased significantly with an increase in the SS content, which indicates that the addition of SS decreased the fluidity of the fresh slurry. The detrimental effect of SS on the flow time and apparent viscosity is mainly due to the increase in the SO_4_^2−^ ion concentration, which competes with anions in the SP, and the SO_4_^2−^ ions are absorbed on the surface of cement particles, resulting in a reduction in the electrostatic repulsion force and steric hindrance effect of SP. Therefore, the dispersibility of fresh slurry was reduced remarkably, and the flow time and apparent viscosity increased.

#### 4.2.3. Bleeding of the UC-Based Slurry

**Figure 8:**
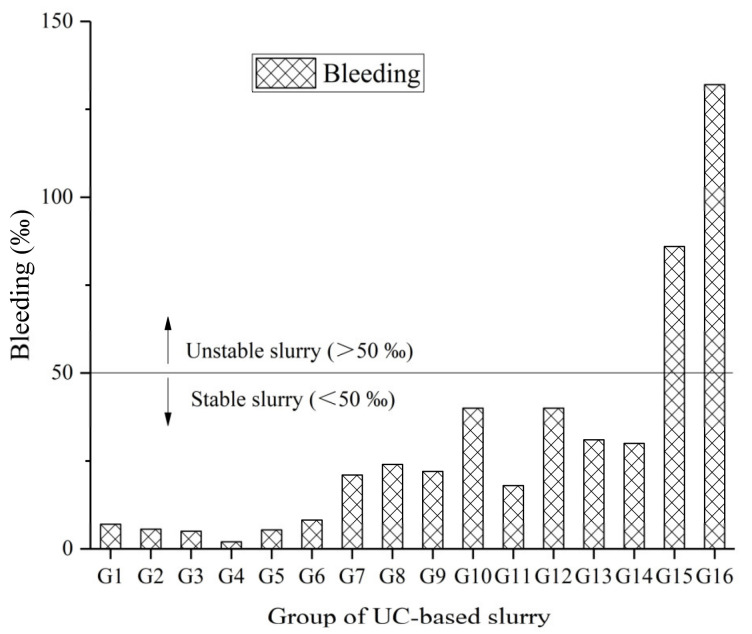
Bleeding of the UC-based slurry.

According to SL62-2014, fresh cement slurry is stable if its bleeding is less than 5% after 2 h from preparation. Because stable slurry can provide a better filling in rock fractures, stable slurry is required for the field grouting process. The results obtained for the bleeding of fresh slurry are shown in Figure 8. Bleeding was observed in most slurries to be less than 5%, which means UC-based slurries are stable in most cases. Mixes G15 and G16 presented the highest bleeding values of 8.6% and 13.2%, while mix G4 presented the lowest bleeding value of 0.2%. This phenomenon may be attributed to the dominant effect of the W/S ratio and SS content as mixes G15 and G16 had a higher W/S ratio and a lower SS content when compared with mix G4.

**Figure 9:**
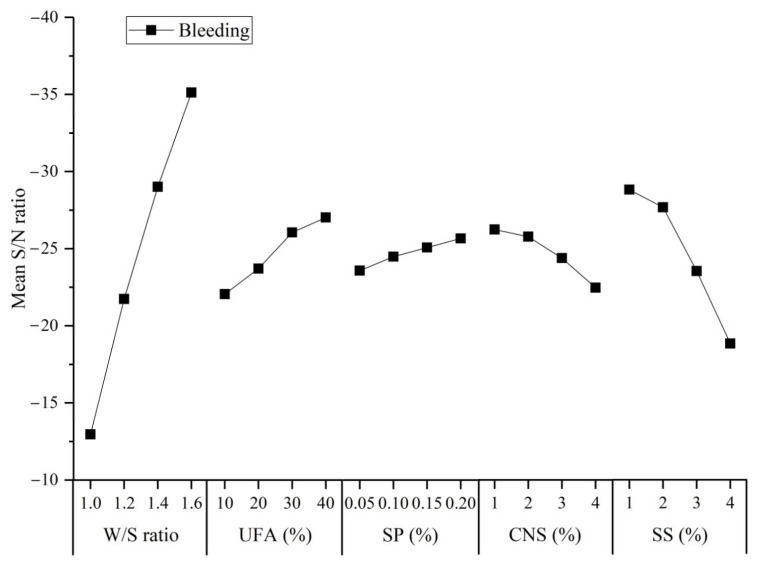
Mean S/N ratio values of the bleeding of the UC-based slurry for different factors.

The S/N ratio of the bleeding calculated by Equation (1) are shown in Table 7. The mean S/N ratio values for bleeding in the UC-based slurry for different factors are presented in Figure 9.

As indicated in Figure 9, the mean S/N ratio of bleeding in the UC-based slurry clearly increased with a small increase in the W/S ratio. The effect of the W/S ratio on bleeding was similar to the flow time and apparent viscosity, as frequently reported in the available literature [26,36]. The increase in the bleeding with an increase in the SP content can be ascribed to more SP being present in the UC-based slurry and causing an enhancement of electrostatic repulsion and space steric hindrance effect. The principle of electrostatic repulsion can be explained as follows: the cations (Ca^2+^) on the surface of the cement flocculation structure will adsorb the anions (–COO^−^) released from the SP. Thus, carboxylic acid ions make the surface of the cement particles carry negative charges, which causes electrostatic repulsion between the cement particles and causes the cement particles to release free water in the flocculation structure. The principle of the space steric hindrance effect can be explained as follows: the adsorption of polycarboxylic acids on the surface of the cement particles is “comb type”, and the adsorption layer is formed on the surface of the gel material. When the polymer molecular adsorption layer approaches, there is a physical space barrier between the polymer molecular chains, which prevents the aggregation of cement particles. As reported in the available literature, the bleeding values of UC-based slurries decrease with an increase in the CNS and SS content. This observation can be ascribed to the increase in the surface area of the reaction media caused by the addition of CNS particles and the faster hydration caused by the addition of SS.

#### 4.2.4. Setting Time of the UC-Based Slurry

**Figure 10:**
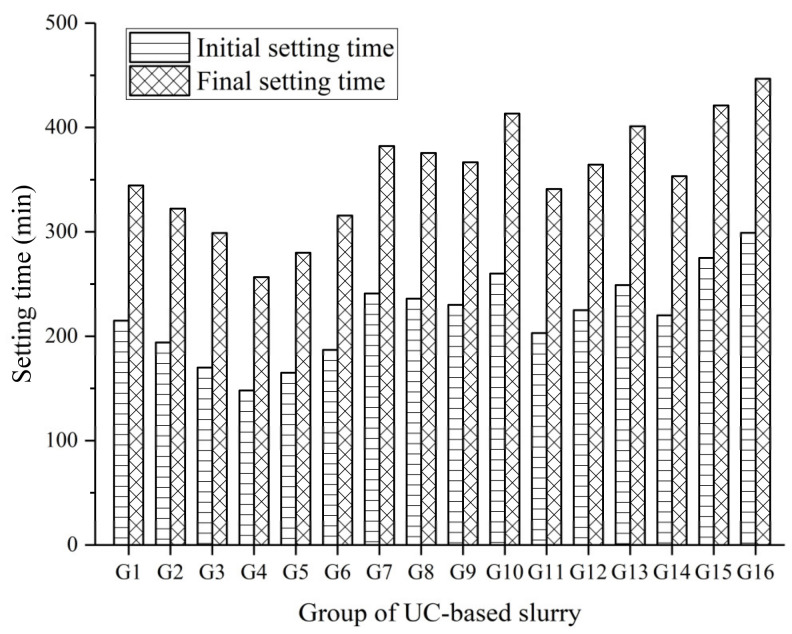
Initial and final setting time of the UC-based slurry.

Based on ASTM Standard C191, the setting time of cement slurry was measured on the sediments after the bleed water is small or negligible. The initial and final setting time of the UC-based slurries are shown in Figure 10.

Figure 10 shows that the initial and final setting times increased as the W/S ratio increased in most cases, as reported in the available literature [11]. In general, the final setting time of a cement-based slurry is required to be within 4–24 h from preparation [32]. This is because a longer setting time can reduce the grouting efficiency and delay the construction process, while a shorter can block the grouting pipe and damage the grouting equipment. The initial setting time of the UC-based slurries ranged from 148 min to 299 min, while the final setting time ranged from 231 min to 402 min. Among all UC-based slurries, group G16 had the longest initial and final setting time due to the higher bleeding. The lowest initial and final setting time of 148 min and 231 min were obtained in group G4, which can be attributed the dominant effect of having the lowest W/S ratio and, to a lesser extent, the highest NS and SS content of all the UC-based slurries.

**Figure 11:**
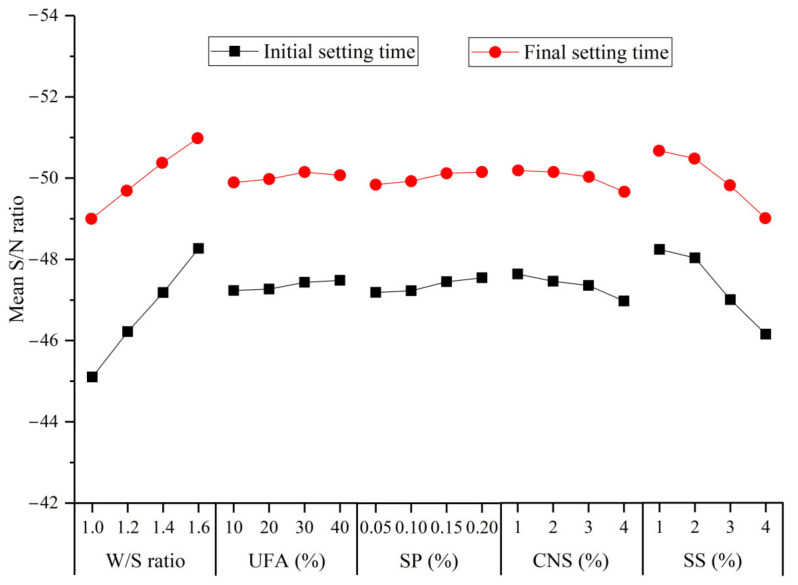
Mean S/N ratio values of the initial and final setting time of the UC-based slurry for different factors.

The S/N ratio of the initial and final setting time calculated by Equation (1) are shown in Table 7. The mean S/N ratio values of the initial and final setting time for UC-based slurries with different factors are presented in Figure 11.

As indicated in Figure 11, the mean S/N ratio of the initial and final setting time increased when the W/S ratio increased from 1.0 to 1.6. The increase in the W/S ratio can cause a decrease in the cement concentration and subsequently slow down the hydration process of the cement. Therefore, the setting time was prolonged. The UFA and SP were also observed to affect the initial and final setting time of UC-based slurries. The UFA provides mainly a filling role in the early hydration stage of the cement and seldom participates in the hydration process of the cement. However, due to the addition of fly ash, the pozzolanic concentration in the slurry is reduced, and the hydration of cement is slowed, which prolongs the setting time. The mechanisms of SP prolonging the setting time in UC-based slurries can be explained as follows: (i) some polycarboxylic acid molecules adsorb on the surface of the cement particles to form insoluble substances, preventing contact between cement particles and water, thus delaying the hydration reaction of the cement; (ii) the carboxyl group of polycarboxylic acid easily forms a complex with Ca^2+^, which reduces the concentration of Ca^2+^ ions in cement slurry and restricts the formation of hydrated calcium silicate (CSH) gel [37]. Therefore, these factors limit the hydration reaction of cement in the early stage and prolong the setting time. However, the S/N ratio decreases with increase in the CNS and SS content indicating a shorter setting time for UC-based slurries. This observation is in good agreement with reports by other researchers [38,39]. The reason for the reduced initial and final setting time at a higher CNS content can be explained as follows: (i) the CNS particles have a higher specific surface area and a higher surface energy, so Ca^2+^ and OH^−^ can easily attach to the CNS particle surfaces, and the reduction of Ca^2+^ and OH^−^ in the slurry accelerated the hydration reaction of the cement; thus, the initial setting time is shortened; and (ii) the rapid consumption of water causes the viscosity of the cement slurry to increases; thus, the solidification of the cement slurry begins earlier. The decrease in setting time with an increase in the SS content can be explained as follows: when the Na_2_SO_4_ solution is mixed with the UC-based slurry, it first reacts with the hydration product (Ca(OH)_2_) to form calcium sulfate dihydrate (CaSO_4_·2H_2_O) and sodium hydroxide (NaOH) (Equation (10)). Then, the calcium sulfate dihydrate reacts quickly with the tricalcium aluminate (C_3_A) in the cement to form AFt (Equation (11)). Thus, the addition of SS accelerates the consumption of cement hydration products leading to a faster hydration process and reducing the initial and final setting time:

Na_2_SO_4_ + Ca(OH)_2_ + 2H_2_O = CaSO_4_·2H_2_O + 2NaOH (11)

3CaSO_4_·2H_2_O + 3CaO·Al_2_O_3_ + 26H_2_O = 3CaO·Al_2_O_3_·3CaSO_4_·32H_2_O (12)

#### 4.2.5. Unconfined Compression Strength of the UC-Based Slurry

**Figure 12:**
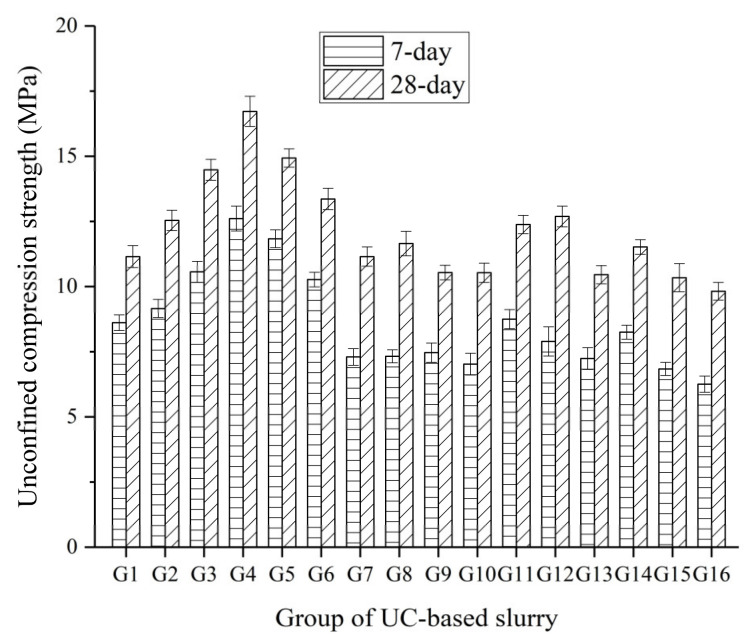
7-day and 28-day UCS of the hardened UC-based slurries.

The 7-day and 28-day unconfined compression strength (UCS) of the hardened UC-based slurries are shown in Figure 12.

As shown in Figure 12, it can be seen that group G4 had the highest UCS values of 12.61 and 16.72 MPa at 7 days and 28 days, respectively. Moreover, group G16 had lowest UCS values of 6.26 and 9.82 MPa at 7 days and 28 days, respectively. This big difference in the UCS of the hardened UC-based slurries at 7 days and 28 days can be ascribed to the significant effect of the W/S ratio. Additionally, UC-based slurries containing a higher SS content was observed to exhibited relatively higher UCS values both at 7 days and 28 days compared with those slurries containing a lower SS content. Therefore, the W/S ratio and SS content can be inferred to be significant for UCS development in UC-based slurries.

**Figure 13:**
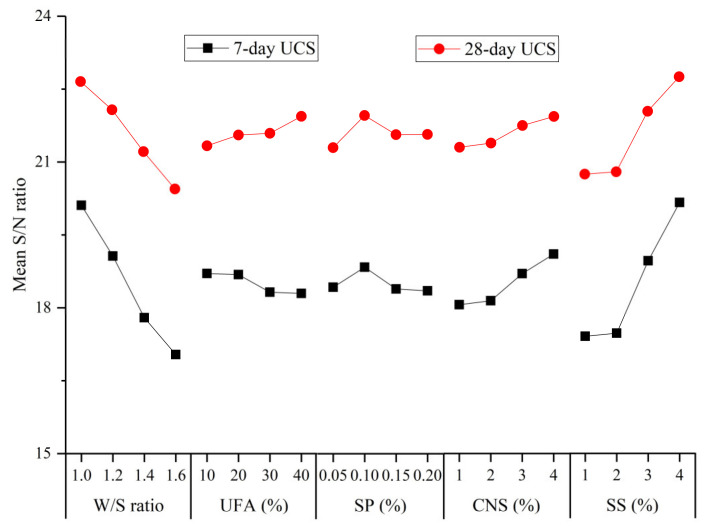
Mean S/N ratio values of the 7-day and 28-day UCS of the hardened UC-based slurries for different factors.

The S/N ratio of the 7-day and 28-day UCS by Equation (3) are shown in Table 7. The mean S/N ratio values of the UCS of the hardened UC-based slurries for different factors are presented in Figure 13.

As indicated in Figure 13, the mean S/N ratio of the 7-day and 28-day UCS of the hardened UC-based slurries decreased significant when the W/S ratio increased from 1.0 to 1.6. The mean S/N ratio of the 7-day UCS decreased with increase in the UFA content, while the mean S/N ratio of the 28-day UCS increased with an increase in the UFA replacement level. Similar observations have been reported by other researchers [40]. At an early age (7 days), the addition of UFA reduces the relative proportion of cement in the UC-based slurry. Then, the hydration product of the cement decreases, which leads to a decrease in the early strength development. However, at a later age (28 days), with the hydration reaction of the cement, the C–S–H gel in the slurry increases, which can cover the surface of UFA particles and provide an alkaline environment. The silica (SiO_2_) and aluminum oxide (Al_2_O_3_) components in the UFA can react with calcium hydroxide (Ca(OH)_2_) to form C–S–H gel and calcium aluminate gel, which contribute to UCS strength development in hardened UC-based slurries at a later age. The variation in the mean S/N ratio of the UCS with the SP content did not show a uniform law at 7 or 28 days, as indicated in Figure 13. This irregular variation may be attributed to the fact that both the 7- and 28-day UCS of hardened UC-based slurries mainly depended on the W/S ratio and SS content, while the effect of SP on the UCS is not significant.

As indicated in Figure 13, the mean S/N ratio of the 7- and 28-day UCS increased with an increase in the CNS content. Some researchers have reported the similar results [41]. The mechanisms of CNS improving the UCS of hardened UC-based slurries can be explained as follows: (i) CNS particles can fill the interstitial spaces between the cement particles, which is helpful in the strength development of the hardened slurry due to the reduction of porosity; and (ii) CNS can react with a cement hydration product (Ca(OH)_2_) to form C–S–H gel, which consumes Ca(OH)_2_ and accelerates the hydration process of cement and, thus, improves the UCS of the hardened UC-based slurries. The mean S/N ratio of the 7- and 28-day UCS was observed to increase significantly with an increase in the SS content. The Na_2_SO_4_ in the SS solution can react with the cement hydration product, Ca(OH)_2_, to form calcium sulfate dihydrate (CaSO_4_·2H_2_O). At the same time, CaSO_4_·2H_2_O can quickly react with C_3_A to form AFt. Moreover, the addition of the SS solution can reduce the concentration of Ca(OH)_2_ and accelerate the hydration process of the cement. Therefore, the addition of SS improved the UCS of the hardened UC-based slurries.

#### 4.2.6. Multi-Response Optimization of the Experimental Results Using the Taguchi Based Grey Relational Analysis Method

In the above analysis and discussion, the optimal levels of various additives corresponding to different properties of UC-based slurries can be obtained. However, a set of levels that can optimize all attributes cannot be obtained. Therefore, in this research, the Taguchi based gray relational analysis method was adopted to obtain a set of optimized level for different properties. The data processing for the Taguchi-Grey relational analysis method is described below.

Step 1: The S/N ratio was normalized using Equations (4) and (6). In this step, different properties of UC-based slurries were standardized to ensure that each property had the same degree of impact. It should be noted that the viscosity, flow time, bleeding and setting time were described as “lower the better” (using the Equation (4)), while UCS was described as “higher the better” (using Equation (6)). The normalized values obtained are presented in Table 8.

Step 2: Based on the results of the normalized values, the mass loss functions were calculated using Equation (7). The results of the mass loss function values are presented in Table 9.

Step 3: According to the results obtained from step 2, the *GRC* values were calculated using Equation (8). Then, the *GRC* values for all tests were converted into a single gray relational grade. The gray relational grade (*GRG*) values were calculated using Equation (9), and the *GRC* and *GRG* values are presented in Table 10.

As shown in Table 10, group G4 (W/S ratio of 1.0, 30% UFA, 0.2% SP, 4% NS and 4% SS) exhibited the highest *GRG* value of 0.810, as G4 had the lowest bleeding and initial and final setting time and the highest UCS of all the UC-based slurries. However, it may not be the best mix due to its poor fluidity (higher flow time and apparent viscosity). Therefore, to obtain the optimum UC-based slurry combination, the average *GRG* for every level of all the parameters was calculated. The optimal level among all the levels of the parameter is defined as the level corresponding to the maximum average *GRG* because it has the greatest effect on the responses. The average *GRG* values for all parameters are shown in Table 11, and the optimal level for each factor is marked (*).

As shown in Table 11, the following optimal combination for a UC-based slurry (corresponding to G4) obtained the highest average *GRG* values: W/S ratio of 1.0, 40% UFA, 0.2% SP, 4% CNS and 4% SS.

#### 4.2.7. Analysis of Variance (ANOVA)

**Figure 14:**
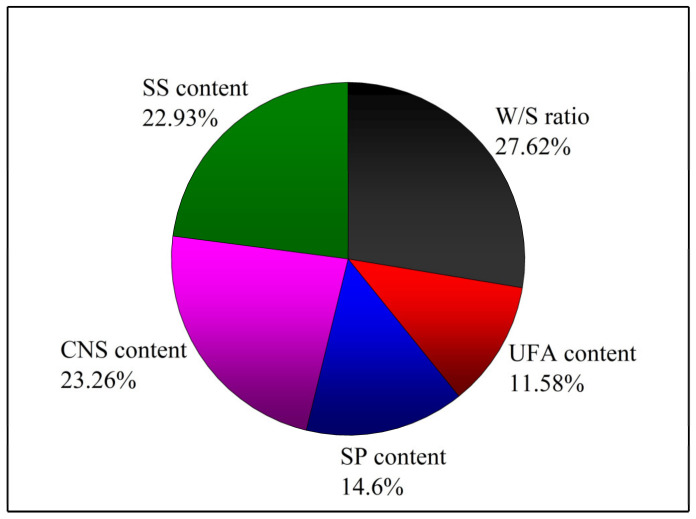
Percentage contributions of the five factors on the tests.

To evaluate the importance of how changing the factors affected the tests, analysis of variance was conducted on the results of the *GRG* values to obtain the percentage contribution of the five factors on the viscosity, flow time, bleeding, setting time and UCS for the UC-based slurries tested in this study. Table 12 shows the ANOVA results of the *GRG* for all the factors. The percentage contributions of the five factors on the tests are presented in Figure 14.

As shown in Table 12 and Figure 14, the W/S ratio was observed to be the most important factor when compared with other factors as it exhibited the highest contributing effect (27.62%) followed by the CNS (23.26%), SS (22.93%), SP (14.6%) and UFA content (11.58%).

#### 4.2.8. Microstructure Analysis of the Hydration Products by SEM

**Figure 15:**
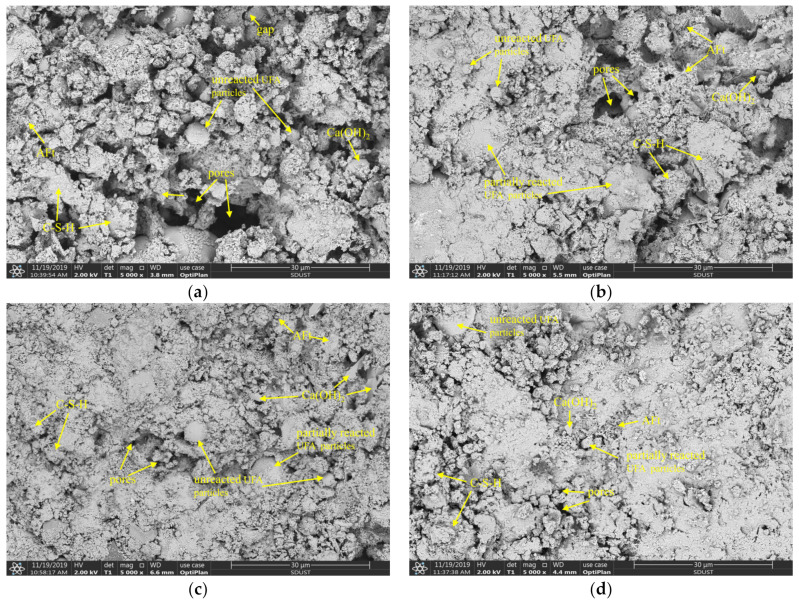
SEM images of 7-day hardened UC-based slurry: (**a**) G1; (**b**) G2; (**c**) G3; (**4**) G4.

In the sixteen UC-based slurry groups, the four groups with the highest gray relational grade values were selected for hydration product microstructure analysis. To better analyze the influence of four admixtures content on the microstructure of the hydration products, the G5, which had the second highest GRG value, was replaced by the G3 because G3 presented satisfactory viscosity compared to group G5 and also had a lower fresh slurry flow time. Therefore, groups G1, G2, G3 and G4 with GRG values of 0.498, 0.536, 0.589 and 0.810, respectively, were chosen for microstructure analysis. Figure 15 shows the SEM images of UC-based slurries (G1, G2, G3 and G4) after 7 days of hydration.

As shown in Figure 15a,b, the hydration process of the UC-based slurries was insufficient, and the microstructure of hydration products was loose and porous after curing for 7 days. Moreover, some unreacted UFA particles and pores existed in the internal microstructure of the hardened slurry. In this hydration stage, UFA particles seldom take part in the hydration reaction and mainly play the role of physical filling. Therefore, the unreacted UFA particles and the presence of pores and gaps resulted in a lower 7-day UCS of the hardened UC-based slurries. Additionally, SiO_2_ from UFA particles was observed to react with the hydration product, Ca(OH)_2_, and generate a small amount of C–S–H gel that adhered to the surface of the UFA particles. Therefore, at an early age (7 days), the presence of UFA in the UC-based slurries delayed the generation of the C–S–H gel. For G2 cured for 7 days, the macropores in the internal microstructure of the hardened UC-based slurry almost disappeared, while some small pores were also observed, which indicated that G2 presented a better microstructure when compared with G1. Additionally, a small number of needle-like AFt crystals were observed within the hardened slurry, which may be due to the addition of SS and SP. These needle-like AFt microstructures overlap and form a porous structure extending to the edge of the pores, resulting in more dense space in the hardened slurry. Therefore, the improvement in the UCS of the hardened slurry can be inferred to be due to the formation of needle-like AFt microstructures filling the pores of the hardened slurry.

As shown in Figure 15c,d, the hydration reaction degree of the UC-based slurries was high, the gap between hydration products was small, and the microstructure of the main binding phase was dense all of which indicate that more CSH gels were generated and were beneficial for the UCS improvement of the hardened slurry. The microstructure improvement of the hardened slurry can be attributed to the addition of CNS and SS. Specifically, CNS can react with Ca(OH)_2_ to generate more C–S–H gels due to its high pozzolanic activity. The physical filling function and interlocking effect of CNS particles can reduce the existence of pores in the microstructure and can optimize the pore distribution in the hardened slurry. Moreover, the addition of CNS can not only accelerate the cement hydration process but can also promote the pozzolanic reaction of the fly ash, as reported in the available literature [33]. As shown in Figure 15, the surface of the UFA particles was no longer clean and smooth (compared with Figure 2b), and many hydration products covered the surface of the spherical particles, which indicates that the pozzolanic activity of UFA was stimulated by the addition of CNS and SS. The mechanisms of SS’s pozzolanic effect on the UFA and cement hydration can be explained as follows: (i) the Na^+^ and SO_4_^2−^ ions released from the sodium sulfate (SS) solution improved the concentration of SO_4_^2−^ ions and alkalinity of the hydration system, which accelerated the dissolution of Al_2_O_3_ from the UFA particles to be converted into AlO_2_^−^ ions, then the SO_4_^2−^ ions reacted with the Ca^2+^, OH^−^ and AlO_2_^−^ ions released from the UC-based slurry to accelerate the generation of AFt; (ii) the improvement in the alkalinity caused by SS solution can promote the reaction of CNS to consume Ca(OH^−^)_2_ and can accelerate the hydration process of cement; (iii) because the AFt layer is loose, the Ca^2+^ ions can pass through the AFt network microstructure and react with highly activated minerals (SiO_2_ and Al_2_O_3_), which also consumes Ca(OH^−^)_2_ and accelerates the hydration process of the cement; and (iv) SO_4_^2−^ ions can replace the SiO_4_^4−^ in the calcium silicate hydrate (C–S–H) gel, and these free SiO_4_^4−^ ions can react with the outer layer Ca^2+^ ions to form C–S–H gel. All of these mechanisms can promote the hydration process of the cement and the pozzolanic effect of the UFA.

#### 4.2.9. MIP Analysis of the Pore Structure of the Hardened UC-Based Slurry

**Figure 16:**
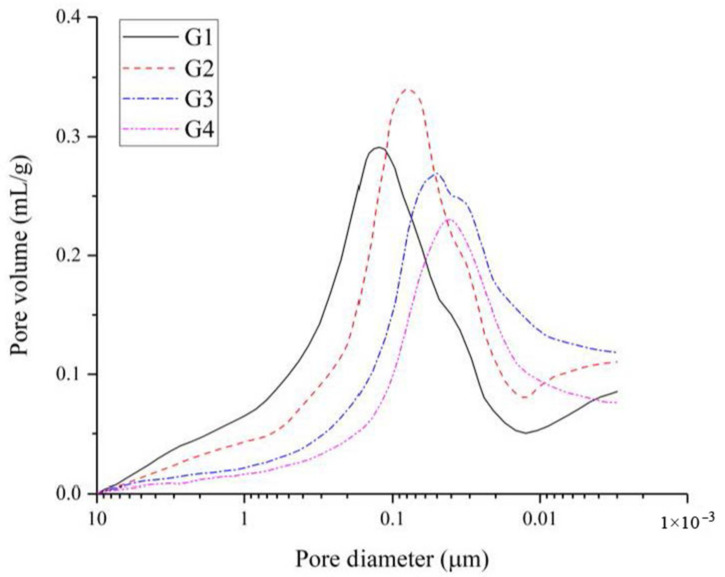
Pore size distribution of hardened slurry (G1, G2, G3, G4) at 7 days.

Quantitative evaluation of the pore size distribution of the hardened slurry (G1, G2, G3, G4) was obtained from the MIP test and the results are shown in Figure 16.

As shown in Figure 16, the pore diameter of the hardened slurry was refined due to the addition of CNS and SS. A decreasing trend of the large capillary pores of the hardened slurry can be observed when more CNS particles and SS were incorporated. The micropores in the hardened slurry were mainly caused by the air introduced in the process of sample preparation. With an increase in the CNS and SS content, the accumulative pore volume decreased, and the refinement of the pore structure of the hardened slurry improved. When the CNS and SS contents were 1%, 2%, 3% and 4%, the pore diameters corresponding to the peak value were 123 nm, 80 nm, 5 0 nm and 43 nm, respectively. Wu [42] divided the pore size into four categories based on different effects of the pore size on concrete performance: harmless pore (pore diameter < 20 nm), few-harm pores (20 nm < pore diameter < 50 nm), harmful pores (50 nm < pore diameter < 200 nm) and multiharm pores (pore diameter > 200 nm). Therefore, with an increase in the CNS and SS content, harmful pores in the hardened slurry decreased, while the harmless and few-harm pores increased. This observation indicates that the CNS particles seem to act as the fillers in the hardened slurry. The mechanism of micropores refinement can be explained as follows: (i) the nanoparticles in the CNS can physically fill the voids in the cement particle skeleton, resulting in an increase in the density and an improvement in the microstructure of the hardened UC-based slurries; (ii) the nanoparticles in the CNS can react quickly with Ca(OH^−^)_2_ to generate C–S–H gels and, then, act as seeds to promote the precipitation and formation of hydration products; these hydration products gradually fill into the space, which decreases the porosity and improves the microstructure of the hardened UC-based slurries. The total porosity results of the hardened UC-based slurries (G1, G2, G3, G4) are summarized in Table 13.

Table 13 shows that the total porosity of 7- and 28-day hardened slurry decreased with an increase in the CNS and SS content. It is clearly seen that with an extension of the curing age, the total porosity of the samples decreased significantly due to the continuous enhancement of the slurry properties with curing age. When comparing the total porosity of slurries with different CNS and SS contents, the slurry with 4% CNS and 4% SS showed best in reducing the total porosity in the long term. Similar observations have been reported in the available literature [38]. With the addition of CNS and SS, the hydration process of the slurry was accelerated, so more hydration products were generated to fill the pores, thus reducing the total porosity and improving the pore size distribution of the hardened UC-based slurries.

## 5. Conclusions

The following conclusions can be drawn from the present study:

(1) The orthogonal test results showed that the degree of influence on the properties of UC-based slurries was as follows: W/S ratio > CNS content > SS content > SP content > UFA content.
(2) For the rheological curves of UC-based slurry, prepared from 0 min to 60 min, can be described as Bingham fluid due to the correlation coefficients are 0.99 in all cases indicating a well fitting of Bingham model to test data.
(3) The addition of CNS and SS showed positive effects on bleeding, setting time and compressive strength of the UC-based slurries, while reducing the fluidity of fresh slurry. However, the detrimental effects of CNS and SS on the fluidity of slurry can be negated by adding UFA and SP.
(4) The SEM test results showed that the microstructure improvement of the hardened slurry can be attributed to the addition of CNS and SS. Moreover, many hydration products covered the surface of the UFA spherical particles, which indicates that the pozzolanic activity of UFA was stimulated by the addition of CNS and SS.
(5) The MIP test results indicated that the pores of the hardened slurry were refined due to the addition of CNS and SS. For the slurry with CNS addition, the volume of harmful and multi-harm pores was significantly reduced. The decrease in total porosity is due to the physical filling effect of the fly ash and the acceleration of the cement’s hydration process.
(6) The results of the Taguchi-Grey relational analysis showed that the optimal mix proportion of the UC-based slurry was G4: W/S ratio of 1.0, 40% UFA, 0.2% SP, 4% CNS and 4% SS.

## Figures and Tables

**Table 1:** Chemical composition and physical properties of the UC and UFA.

Chemical Composition	UC (wt%)	UFA (wt%)
CaO	62.51	3.98
SiO_2_	21.53	40.55
Al_2_O_3_	4.08	17.83
Fe_2_O_3_	2.89	28.98
MgO	3.31	1.56
Na_2_O	0.21	0.98
K_2_O	0.57	1.43
TiO_2_	0.30	0.93
SO_3_	3.04	1.32
LOI	1.56	2.44
Average particle size	5.06 µm	3.56 µm
Specific surface (m^2^/kg)	920	1033

**Table 2:** Properties of the CNS (in liquid form).

Aspect	Specific Gravity	pH	SiO_2_ (%)	Na_2_O (%)	Average Particle Size (nm)
milky white	1.2	10	30	0.34	30

**Table 3:** Five factors and four levels of orthogonal test design.

Level	Factors				
	W/S Ratio	UFA Content (%)	SP Content (%)	CNS Content (%)	SS Content (%)
1	1.0	10	0.05	1	1
2	1.2	20	0.1	2	2
3	1.4	30	0.15	3	3
4	1.6	40	0.2	4	4

**Table 4:** The orthogonal test scheme L16(4^5^).

Group	W/S Ratio	UFA Content (%)	SP Content (%)	CNS Content (%)	SS Content (%)
G1	1.0	10	0.05	1	1
G2	1.0	20	0.1	2	2
G3	1.0	30	0.15	3	3
G4	1.0	40	0.2	4	4
G5	1.2	10	0.1	3	4
G6	1.2	20	0.05	4	3
G7	1.2	30	0.2	1	2
G8	1.2	40	0.15	2	1
G9	1.4	10	0.15	4	2
G10	1.4	20	0.2	3	1
G11	1.4	30	0.05	2	4
G12	1.4	40	0.1	1	3
G13	1.6	10	0.2	2	3
G14	1.6	20	0.15	1	4
G15	1.6	30	0.1	4	1
G16	1.6	40	0.05	3	2

**Table 5:** Gradation of UC and UFA.

Materials	Grain Sizes (µm)				Specific Surface (m^2^/kg)
	*d*_max_	*d*_95_	*d*_50_	*d*_10_	
UC	17.46	12.49	5.06	1.1	920
UFA	13.74	7.28	3.56	0.9	1033

**Table 6:** Rheological parameters of the fresh slurry.

Test Time (min)	*τ*_0_ (Pa)	*η* (mPa·s)	*R* ^2^
0	2.28	23	0.99
30	2.77	37	0.99
60	3.45	48	0.99

**Table 7:** S/N ratio values of properties of the UC-based slurry.

Group	Flow Time	Apparent Viscosity	Bleeding	Initial Setting Time	Final Setting Time	7-Day UCS ^1^	28-Day UCS ^1^
G1	−26.42	−37.92	−16.90	−48.46	−49.83	18.71	20.95
G2	−26.56	−40.05	−14.96	−47.75	−49.25	19.24	21.97
G3	−29.44	−43.85	−13.98	−46.85	−48.60	20.48	23.22
G4	−31.06	−45.95	−6.02	−45.93	−47.27	22.01	24.46
G5	−29.85	−45.46	−14.65	−46.65	−48.03	21.46	23.48
G6	−28.55	−43.03	−18.28	−47.49	−49.07	20.23	22.52
G7	−24.53	−29.37	−26.44	−49.28	−50.73	17.27	20.95
G8	−24.58	−30.05	−27.60	−49.13	−50.58	17.30	21.33
G9	−24.72	−32.00	−26.85	−48.94	−50.37	17.46	20.46
G10	−23.88	−26.19	−32.04	−49.83	-51.41	16.93	20.45
G11	−26.71	−39.56	−25.11	−48.06	−49.74	18.84	21.85
G12	−24.79	−32.38	−32.04	−48.79	−50.32	17.95	22.07
G13	−24.06	−28.76	−29.83	−49.51	−51.15	17.19	20.39
G14	−25.64	−34.12	−29.54	−48.63	−50.05	18.33	21.23
G15	−23.82	−25.34	−38.69	−50.24	−51.57	16.70	20.29
G16	−23.66	−23.92	−42.41	−50.86	−52.08	15.92	19.84

^1^ UCS: unconfined compression strength.

**Table 8:** Normalized values of different properties of the UC-based slurry.

Group	Flow Time	Apparent Viscosity	Bleeding	Initial Setting Time	Final Setting Time	7-Day UCS	28-Day UCS
G1	0.63	0.36	0.70	0.47	0.47	0.46	0.24
G2	0.61	0.27	0.75	0.62	0.59	0.54	0.46
G3	0.22	0.10	0.78	0.80	0.72	0.75	0.73
G4	0.00	0.00	1.00	1.00	1.00	1.00	1.00
G5	0.16	0.02	0.76	0.85	0.84	0.91	0.79
G6	0.34	0.13	0.66	0.67	0.63	0.71	0.58
G7	0.88	0.75	0.44	0.31	0.28	0.22	0.24
G8	0.88	0.72	0.41	0.34	0.31	0.23	0.32
G9	0.86	0.63	0.43	0.37	0.36	0.25	0.13
G10	0.97	0.90	0.28	0.20	0.14	0.17	0.13
G11	0.59	0.29	0.48	0.55	0.49	0.48	0.44
G12	0.85	0.62	0.28	0.40	0.37	0.33	0.48
G13	0.95	0.78	0.35	0.26	0.19	0.21	0.12
G14	0.73	0.54	0.35	0.44	0.42	0.40	0.30
G15	0.98	0.94	0.10	0.12	0.11	0.13	0.10
G16	1.00	1.00	0.00	0.00	0.00	0.00	0.00

**Table 9:** Mass loss function values of different properties of the UC-based slurry.

Group	Flow Time	Apparent Viscosity	Bleeding	Initial Setting Time	Final Setting Time	7-Day UCS	28-Day UCS
G1	0.37	0.64	0.30	0.53	0.53	0.54	0.76
G2	0.39	0.73	0.25	0.38	0.41	0.46	0.54
G3	0.78	0.90	0.22	0.20	0.28	0.25	0.27
G4	1.00	1.00	0.00	0.00	0.00	0.00	0.00
G5	0.84	0.98	0.24	0.15	0.16	0.09	0.21
G6	0.66	0.87	0.34	0.33	0.37	0.29	0.42
G7	0.12	0.25	0.56	0.69	0.72	0.78	0.76
G8	0.12	0.28	0.59	0.66	0.69	0.77	0.68
G9	0.14	0.37	0.57	0.63	0.64	0.75	0.87
G10	0.03	0.10	0.72	0.80	0.86	0.83	0.87
G11	0.41	0.71	0.52	0.45	0.51	0.52	0.56
G12	0.15	0.38	0.72	0.60	0.63	0.67	0.52
G13	0.05	0.22	0.65	0.74	0.81	0.79	0.88
G14	0.27	0.46	0.65	0.56	0.58	0.60	0.70
G15	0.02	0.06	0.90	0.88	0.89	0.87	0.90
G16	0.00	0.00	1.00	1.00	1.00	1.00	1.00

**Table 10:** *GRC* values of different properties of the UC-based slurry.

Group	*GRC* Values							Grey Relational Grade
	Flow Time	Apparent Viscosity	Bleeding	Initial Setting Time	Final Setting Time	7-Day UCS	28-Day UCS	
G1	0.573	0.440	0.626	0.485	0.485	0.480	0.397	0.498
G2	0.561	0.406	0.670	0.565	0.549	0.523	0.481	0.536
G3	0.390	0.356	0.696	0.718	0.645	0.665	0.650	0.589
G4	0.333	0.333	1.000	1.002	1.000	1.001	1.002	0.810
G5	0.374	0.338	0.678	0.764	0.761	0.848	0.702	0.638
G6	0.431	0.366	0.598	0.601	0.573	0.631	0.543	0.535
G7	0.809	0.669	0.471	0.419	0.410	0.391	0.397	0.509
G8	0.800	0.643	0.457	0.430	0.421	0.392	0.424	0.510
G9	0.778	0.577	0.466	0.444	0.437	0.401	0.366	0.496
G10	0.943	0.829	0.412	0.384	0.367	0.375	0.365	0.525
G11	0.549	0.413	0.488	0.527	0.493	0.490	0.470	0.490
G12	0.767	0.566	0.412	0.456	0.441	0.428	0.491	0.509
G13	0.903	0.695	0.433	0.403	0.383	0.387	0.362	0.509
G14	0.651	0.519	0.436	0.470	0.464	0.453	0.417	0.487
G15	0.958	0.886	0.358	0.362	0.359	0.365	0.357	0.521
G16	1.000	1.000	0.333	0.333	0.333	0.333	0.333	0.524

**Table 11:** Average *GRG* values for each level of all the factors.

Factor	Level 1	Level 2	Level 3	Level 4
W/S ratio	0.608 *	0.548	0.505	0.510
UFA content	0.535	0.521	0.527	0.588 *
SP content	0.512	0.551	0.520	0.589 *
CNS content	0.501	0.511	0.569	0.590 *
SS content	0.513	0.516	0.535	0.606 *

*: optimal level for each factor.

**Table 12:** ANOVA results of *GRG* for the factors.

Factor	DOF ^1^	SOS ^2^	MS ^3^	Percentage Contribution (%)
W/S ratio	3	0.0272	0.0091	27.62
UFA content	3	0.0114	0.0038	11.58
SP content	3	0.0144	0.0048	14.6
CNS content	3	0.0229	0.0076	23.26
SS content	3	0.0226	0.0075	22.93
Error	-	-	-	-
Total	15	0.0985		100

^1^ DOF: Degree of freedom; ^2^ SOS: Sum of squares; ^3^ MS: Average squares.

**Table 13:** Total porosity of the hardened UC-based slurry at 7 and 28 days.

Group	7-Day	28-Day
G1	21.8	17.9
G2	19.7	16.8
G3	16.2	13.4
G4	15.3	12.7

## Data Availability

The data presented in this study are available on request to the corresponding author.
